# Effects of Pelletized and Coated Organic Fertilizers on Flavor Compounds of Tomato Fruits and Leaves

**DOI:** 10.3390/foods13111653

**Published:** 2024-05-25

**Authors:** Huiying Jiao, Sijia Wu, Jingming Li, Yanxin Sun

**Affiliations:** 1 Faculty of Food Science and Engineering, China Agricultural University-Sichuan Advanced Agricultural & Industrial Institute, Chengdu 611430, China; jiao2303014957@163.com; 2 Faculty of Food Science and Engineering, China Agricultural University, Beijing 100091, China; wusijia.hi@163.com; 3 Institute of Plant Nutrition, Resource and Environment, Beijing Academy of Agriculture and Forestry Sciences, Beijing 100097, China

**Keywords:** tomato fruit, organic fertilizer, pelletizing and coating, volatile compounds, flavor

## Abstract

The application of organic fertilizers is one of the most important agricultural measures aimed at improving the flavor and productivity of *Lycopersicon esculentum*, with the granulation and coating of organic fertilizers, which can reduce seepage losses of great significance to the ecosystem. In this study, Jingcai 8 tomato was selected as the test material. Headspace solid-phase microextraction and gas chromatography–mass spectrometry (HS-SPME-GC-MS) methods were used to investigate the effects of different pelletized organic fertilizers and various coating materials on the flavor profile of the tomatoes. The results indicated that 67 volatile organic compounds (VOCs) were identified in the tomato fruits and 62 volatile compounds were identified in the leaves under different fertilizer treatments. The volatile compound content of the fruits in the BP treatment group was 35.38 μg/g, which was higher than that in other treatment groups, and the volatile compound content of the leaves was lower. A differential compound analysis with log_2_|fold change| ≥ 1 and variable important in projection (VIP) > 1 highlighted styrene, 3-methyl-1-butanol, and (E, E)-2,4-hexadienal as the major up-regulated compounds and methyl salicylate as the major down-regulated compound in the tomato fruit BCK (control) vs. BP. Moreover, the α-phellandrene content decreased in the tomato leaves. In addition, an analysis of the tomato fruit differential compounds and compounds with odor activity values (OAV) of ≥ 1, considering the OAV values of characteristic aroma compounds, identified key compounds affecting the flavor of the tomato fruits under the BP treatment. These included 2-nonenal, (E)-2-pentylfuran, trans-β-ionone, 1-penten-3-one, (E, E)-2,4-hexadienal, and 3-hexenol (fruity, floral, and herbaceous odors), (E, E)-2,4-heptadienal (fatty odor), and hexanal (green odor). The combined results analysis of the volatile compound content, differential compounds, and OAV values of characteristic aroma compounds aimed to clarify that the BP treatment group, which applied pelletized, large-grain organic fertilizer with polyurethane (pozzolanic + small-grain oil-coated + 2% paraffinic + 4% polyurethane) as a coating material, proved to be most effective in influencing the flavor of the tomato fruits. This finding lays the foundation for its potential commercial application in artificial orchards.

## 1. Introduction

*Lycopersicon esculentum* belongs to the genus *Lycopersicon*, within the Solanaceae Juss. family, ranking as the third-largest vegetable and the tenth-largest economic product globally. In 2019, its annual production reached approximately 180,766,329 tons, reflecting its widespread consumption [1]. The huge demand for tomato fruits stems from their classification as a functional food serving as a vital source of essential nutrients for a healthy human diet. Consumers prioritize their nutritional and aromatic qualities when making purchasing decisions [2]. To date, over 400 volatile metabolites have been identified in tomato fruits, mainly alcohols, aldehydes, ketones, esters, sulfur, acyclic, cyclic and heterocyclic hydrocarbons, free acids, phenols, and oxo-compounds, among which, 28 major volatiles contribute to the characteristic flavor of tomatoes [3,4]. Compounds such as hexanal, (Z)-3-hexenal, (Z)-3-hexen-1-ol, 2-phenylethanol, methyl salicylate, and 6-methyl-5-hepten-2-one play pivotal roles in enhancing the green, floral, and fruity flavor profile of tomatoes. These compounds are mainly derived from fatty acids, carotenoids, and amino acids [5]. Moreover, the core flavor of the tomato fruit offers various health benefits, including reductions in cancer, cardiovascular diseases, neurodegenerative diseases, and antibacterial effects [6]. For example, lycopene, a prominent bioactive compound found in tomatoes, exhibits protective properties against cardiovascular disease and various cancers such as prostate, breast, lung, colorectal, endometrial, oral, and gastric cancers. Notably, 6-methyl-5-hepten-2-one, a flavoring substance derived directly from lycopene, along with β-ionone, a β-carotene-derived product, and geranylacetone are likely to originate from lycopene-related volatiles [7]. In addition, 2-isobutylthiazole contributes to the characteristic aroma associated with tomatoes, while guaiacol and methyl salicylate impart odors that are less favored by consumers.

In recent years, research on high-demand crops has focused on enhancing yield and resistance, often neglecting organoleptic quality and nutritional value [8,9]. The nutritional health value and flavor of tomatoes are primarily determined by their chemical composition, which, in turn, is influenced by the cultivation environment and agronomic factors such as variety, climate, soil quality and fertilizer application [10,11]. In particular, the type of fertilizer applied directly affects the fruit’s bioactive compounds and mineral content, creating complex links in balancing the senses [12]. While conventional chemical fertilizers are preferred for their cost effectiveness and rapid nutrient absorption, one study in particular by Akiyama et al. demonstrated that organic fertilizers yield a higher nutritive value when applied to tomatoes [13]. Additionally, research by Muscolo et al. comparing different organic fertilizers on Red Topepo sweet pepper revealed improved nutritional, organoleptic, and economic values [14]. The results of Wang et al. also demonstrated that the application of organic fertilizers led to a broader spectrum of apple varieties with a higher relative content of aromatic compounds compared to chemical fertilizers [15]. Some researchers and scholars have similarly studied the combined application of organic and inorganic fertilizers on fruit aroma compounds, finding that they did not significantly differ from standard chemical fertilization [16]. The results showed that organic fertilizers have a more significant effect on crop growth than chemical fertilizers. They supplement nutrients lacking in the soil, gradually reducing the toxicity of chemical fertilizers and steadily increasing soil fertility, thus enhancing the nutritional, organoleptic, and economic values of crops. In addition, fertilizer application may also impact chlorophyll synthesis and, consequently, the photosynthetic capacity of leaves, which, in turn, influences the production of flavor compounds in fruits through the synthesis of primary and secondary metabolites during photosynthesis [17]. Collectively, these studies indicate that organic fertilizers function as bio-stimulants, exerting a significant influence on the aroma characteristics of fruits.

However, the loss of active ingredients during the application of organic fertilizers affects the nutritional value and organoleptic quality of the fruit and is also a potential source of environmental pollution problems, such as water body eutrophication and groundwater contamination by harmful substances [18]. Thus, the granulation and coating of organic fertilizers to reduce leakage losses are of significant importance to the ecological environment. Currently, while there have been numerous studies on the effects of organic and inorganic fertilizers on tomato fruit quality, fewer studies have focused on tomato flavor quality under different organic fertilizer granulation and coating post-treatments. In this study, tomato (Jingcai 8) grown in the Changping Agricultural Carnival, Beijing, China, was selected as the test material. Two types of organic fertilizers pelletized with different material coatings were applied to treat the tomatoes. HS-SPME-GC-MS methods were used to analyze the characteristics of the volatile components in the ripening stage of the fruit and leaves of the tomato. Considering the nutrient balance of the soil ecosystem and the pursuit of sustainable agricultural development, we investigated the effects of various granulated organic fertilizers and different coating materials on the flavor of the tomato fruits. Our objective was to identify the organic fertilizers and their coating materials suitable for orchard application, in order to improve the quality of tomato fruits.

## 2. Materials and Methods

### 2.1. Sample Information and Preparation

To analyze the effects of different fertilizers on the volatile compounds in tomato fruits and leaves, 200 g of tomato fruits and 10 g of leaves at the ripening stage under various fertilizer treatments were selected as the test materials. In 2021, tomatoes were planted at the Changping Agricultural Carnival (longitude 116°23′ E, latitude 40°22′ N) in Beijing, China. Different fertilizers were applied to the field, with all other cultivation and planting conditions kept constant except for the fertilizers. The samples were collected in September 2022 when the fruits were ripe. Fertilizers were selected from the laboratory configuration and included large-grain (B) and small-grain organic fertilizers (S), both pelletized by the company, as described in (Table S1), and coated separately: O—no coating material, X—corn sugar gum coating (pozzolanic + small-grain oil coating + corn sugar gum + hydroxy-methylcellulose), T—alginate coating (pozzolanic + small-grain oil coating + alginate sugar + biochar + potassium carbonate + hydroxy-methylcellulose), and P—polyurethane coating (pozzolanic + small-grain oil coating + 2% paraffin + 4% polyurethane). The organic fertilizer coating materials were each 15 μm thick, with a release period of approximately 60 days and a coating rate of 3%. The experiment employed control variables, with two areas of the experimental field selected for the application of two organic fertilizers, B and S. Within these areas, BCK and SCK served as the control groups (receiving no fertilizer) for the respective organic fertilizers. Additionally, eight envelope treatments—BO, SO, BT, ST, BX, SX, BP, and SP—comprised the organic fertilizers, which were mixed with the soil for application. The tomato fruits and leaves, free from breakage and disease, were randomly selected for each treatment, and the experiment was replicated three times. The selected samples were rinsed with distilled water, dried with blotting paper, and stored at −80 °C for preservation.

### 2.2. Important Volatile Compound Detection

Detection of the volatile compounds in the fruits: fruit samples were taken for primary crushing under liquid nitrogen, 60 g was weighed, 1.20 g of cross-linked polyvinylpyrrolidone and 0.60 g of d-gluconolactone were added, crushed into powder by a crusher, and then quickly transferred to 50 mL centrifuge tubes and stored in a refrigerator at 4 °C for 5 h. Subsequently, the samples were transferred to a centrifuge tube after the initial 5 h storage period. After 5 h, the samples were removed and centrifuged at 4 °C for 15 min at 8000 r/min. In total, 2 mL of the supernatant was extracted and 2-octanol (14.00 mg/L, 10 μL) was added as the internal standard. After adding l g of NaCl, the sample was transferred to a 15 mL headspace vial, capped, sealed, and equilibrated at 40 °C for 30 min. Subsequently, the gas–liquid phase reached equilibrium, and the sample was adsorbed at 40 °C for 30 min before being manually injected for desorption for 8 min without shunt injection in the solid-phase microextraction. An INNOWAX column (60 m × 0.25 mm × 0.25 μm, Agilent, Santa Clara, CA, USA) was used, with a heating program starting at 40 °C for 3 min, followed by an increase to 200 °C at 4 °C/min, where it was held for 5 min. The carrier gas used was high-purity helium, with a purity of no less than 99.999%, at a flow rate of 1 mL/min. The inlet temperature was maintained at 250 °C, and the temperature of the transfer line was also set at 250 °C. The mass spectrometer operated in electron collision mode at 70 eV, scanning within the range of *m*/*z* 30–350.

Determination of the leaf volatiles: leaf samples were crushed under liquid nitrogen, with 0.5 g weighed and mixed with 5 mL of ultrapure water. The mixture was then transferred into a 15 mL headspace flask and sonicated for 5 min at 70 kHz in an ice-water bath. 2-Nonanone (33.00 mg/L, 10 μL) was added to the treated leaf samples as an internal standard. Following equilibration at 60 °C for 30 min, the samples were adsorbed at 60 °C for an additional 30 min after reaching gas–liquid phase equilibrium, and then manually injected by solid-phase microextraction without shunt injection and desorbed for 8 min. Using an INNOWAX column (60 m × 0.25 mm × 0.25 μm, Agilent, USA), the heating protocol was initiated at 60 °C for 3 min, then increased to 240 °C at 4 °C/min, where it was held for 7 min. The carrier gas employed was high-purity helium with a purity of no less than 99.999% at a flow rate of 1 mL/min. The port temperature of the injection port was set at 250 °C, while the transfer line temperature mirrored this at 250 °C. The temperature of the ion source was 230 °C, and the mass spectrometer operated in electron collision mode at 70 eV with a scanning range of *m*/*z* 30–350.

### 2.3. Qualitative and Quantitative Analysis

The NIST library combined with retention indices was used for characterization and the internal standard method was used for quantification. The retention indices [5] and mass spectral data for the volatiles were calculated using the Automated Mass Deposition and Identification Software (AMDIS) (Version 2.73) and cross-referenced with the NIST 11 standard library. The mass spectral data and retention indices of the target compounds were qualitatively compared with the standards, and their respective standard curves were used for quantification. Compounds lacking standards underwent qualitative comparison with the NIST11 spectral library based on their mass spectral data and retention indices and were quantified using standards with similar chemical structures and carbon atom counts.

### 2.4. Statistical Analysis

All data underwent analysis using the SPSS analysis of variance (ANOVA) software (version 23.0, IBM, Inc., Beijing China) and are presented as mean (±SE; standard error). Combined with an orthogonal partial least squares discriminant analysis (OPLS-DA) to accurately identify differential metabolites, variable important projections (VIP) were obtained based on the OPLS-DA results to preliminarily screen for differential metabolites between groups. This was further complemented by consideration of the multiplicity of differences (Fold Change) to further screen the selection of differential metabolites [19]. Images within the text were analyzed using Origin (2019) and RStudio software (Version 4.1.3). SIMCA 14.1 was used to perform the orthogonal partial least squares discriminant analysis and calculate the predictor VIP. KEGG was used to perform compound functional annotation https://www.kegg.jp/kegg/pathway.html (accessed on 11 December 2023).

## 3. Results

### 3.1. Analysis of Volatile Compounds in Tomato Fruits and Leaves under Different Treatments

**Figure 1:**
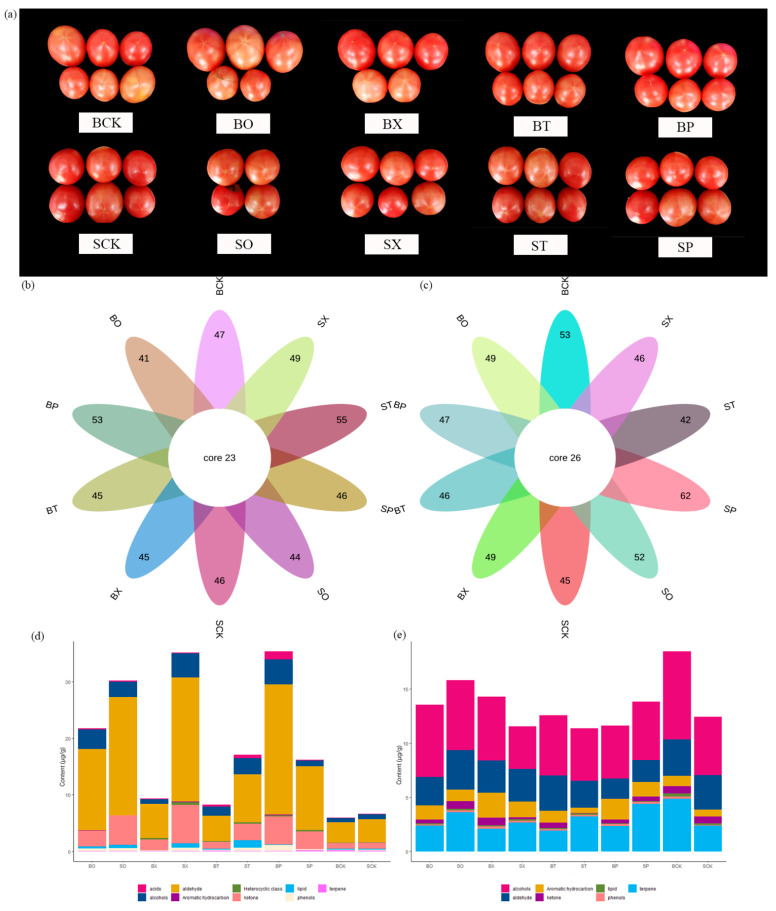
Volatile organic compounds in tomato fruits and leaves of different treatment groups. (**a**)—Ripened fruit samples of different treatment groups; (**b**,**c**)—petalograms showing the quantity of volatile compounds in tomato fruits and leaves of different treatment groups; and (**d**,**e**)—histograms of volatile compounds content in tomato fruits and leaves of different treatment groups. Note: (**b**,**d**)—fruits and (**c**,**e**)—leaves.

The volatile organic compounds’ components, OAV, and relative contents of the tomato fruits (Figure 1a) and leaves were analyzed using headspace solid-phase microextraction (HS-SPME) coupled with gas chromatography–mass spectrometry (GC-MS) under various fertilizer treatments. A total of 67 compounds were detected in the tomato fruits across different fertilizer treatments, with a total of 23 compounds identified across 10 different treatments (Figure 1b). These compounds primarily included alcohols (16), aldehydes (19), ketones (8), oeno-terpenes (6), lipids (7), acids (3), phenols (6), aromatic hydrocarbons (1), and heterocyclics (3) (Figure S1a). Notably, the content of acetic acid butyl ester exhibited a significant and positive correlation with other fertilizer treatment groups, excluding the SP and SO treatment groups, while the methyl salicylate content in fruits was significantly and positively correlated with the treatment groups, except BP and BX. From Figure 1d, aldehyde, alcohols, and ketones emerged as the main components of volatile compounds in the tomato fruits, with higher concentrations observed under the BP and SX treatments, reaching 35.38 μg/g and 35.19 μg/g, respectively. The contents of acids, aldehyde, alcohols, and ketone in the tomato fruits under different treatments showed varying degrees of increase compared to the control, with higher increases in the contents of alcohols, aldehyde, and ketone in the BP, SX, BO, and SO treatment groups.

A total of 62 compounds were detected in the tomato leaves, with 26 compounds common to 10 treatments (Figure 1c). The volatile compounds in the leaves mainly consisted of alcohols (20), aldehydes (13), ketones (6), olefin terpenes (13), lipids (5), phenols (3), and aromatic hydrocarbons (2). Under the ST and SX treatments, the volatile compound correlations were more consistent, showing significant negative correlations with 4-(1-methylethyl)-2-cyclohexen-1-one content and significant positive correlations with the 3-methyl-2-buten-1-ol, (+)-4-carene, and β-phellandrene contents (Figure S1b). Volatile compound correlations were more similar for the BT and SCK treatment groups, with the remaining treatment groups displaying significant positive correlations with the β-phellandrene content and significant negative correlations with 6-methyl-5hepten-2-one and 4-(1-methylethyl)-2-Cyclohexen-1-one content. The volatile compound contents of the tomato fruits varied more than those of the leaves under different treatments, reaching a peak at 18.50 μg/g in the BCK-treated leaves (Figure 1e). BO, BX, BT, and BP showed different increases in aromatic hydrocarbon content and decreases in all other compounds when compared to the control BCK.

**Figure 2:**
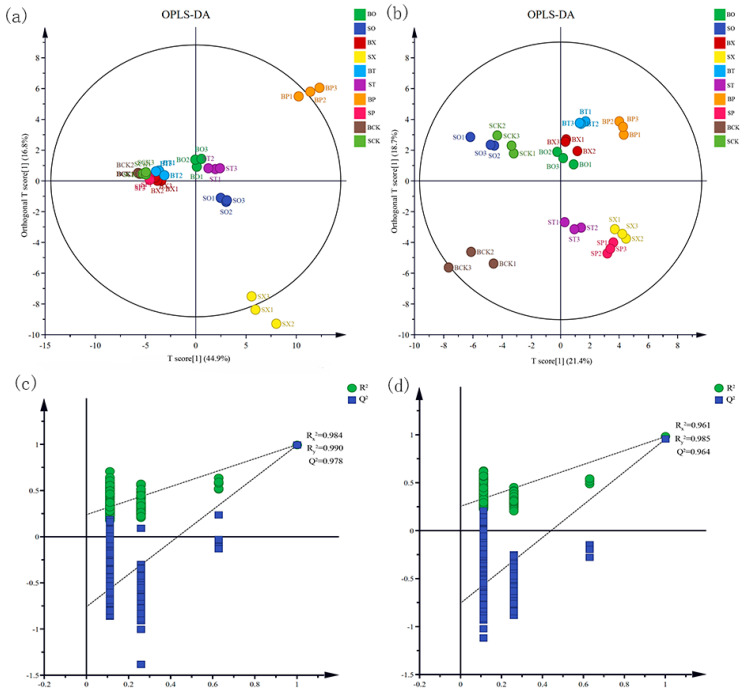
OPLS-DA score plots and model cross-validation for tomato fruits and leaves in different treatments. Note: (**a**,**c**)—fruit and (**b**,**d**)—leaves.

OPLS-DA was used to confirm differences between the fertilizer treatments, revealing the distinct clustering of volatile compound compositions in both the tomato fruits and leaves following different treatments. The two groups of samples, the BP and SX groups, appeared more isolated from the other groups in the fruits, while the tomato leaf compounds under the BCK, ST, SP and SX treatments were more distant from the other treatment groups (Figure 2a,b). The fit indices of the independent variables (R_x_^2^) were 0.984 and 0.961, while those for the dependent variables (R_y_^2^) were 0.990 and 0.985, respectively, in the tomato fruits and leaves. Model prediction indices (Q^2^) were recorded at 0.978 and 0.964, respectively. With R^2^ and Q^2^ exceeding 0.5 and the point of intersection of the Q^2^ regression line with the longitudinal axis falling below 0, the model validation proved effective (Figure 2c,d), thus facilitating the analysis of the volatile compound differences between the tomato fruits and leaves.

### 3.2. Analysis of Different Volatile Compounds in Tomato Fruits and Leaves under Different Treatments

**Figure 3:**
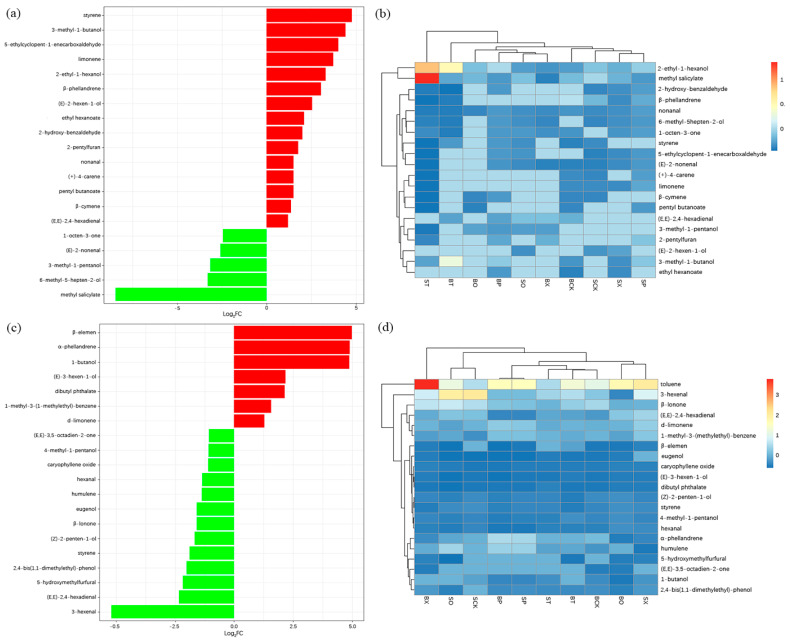
Analysis of tomato fruit and leaf differential volatile metabolites. (**a**,**c**)—Fold difference histograms of tomato fruit and leaf differential volatile compounds (top 20) and (**b**,**d**)—normalized clustered heatmap of tomato fruit and leaf differential volatile UVS chemotaxis (top 20). Note: (**a**,**b**)—fruit; (**c**,**d**)—leaf; red represents up-regulated differential compounds, green represents down-regulated differential compounds.

Combined with the criteria of log_2_|FC| ≥ 1 and VIP > 1, the differential compounds of the tomato fruits and leaves under 10 fertilization treatments were screened to compare the fold changes of the differences in the contents of the differential compounds in each fraction. The results, showcasing the top 20 differential compounds, are shown in Figure 3a,c. Among the 15 up-regulated compounds in the fruit, styrene and 3-methyl-1-butanol exhibited significant fold changes, while methyl salicylate, the down-regulated compound, displayed the most substantial change in content. In the tomato leaves, seven up-regulated compounds, including β-elemen, α-phellandrene, and 1-butanol, showed significant variations, whereas the down-regulated compound, 3-hexenal, demonstrated notable differences in content. Based on the normalized treatments, it was observed that the relative contents of methyl salicylate and 2-ethyl-1-hexanol in the tomato fruits were higher under the ST treatment (Figure 3b). Except for the SO and SCK treatments, the relative content of toluene was higher, with the highest observed under the BX treatment (Figure 3d).

The three treatment groups SO, SX, and BP, characterized by higher contents of volatile compounds in the tomato fruits, were selected for a differential compound analysis alongside their aroma substance content. BCK vs. BP had a total of 18 differential compounds (up-regulated compound **6**, down-regulated compound **1**), SCK vs. SO had a total of 13 differential compounds (up-regulated compound **5**), and SCK vs. SX had a total of 16 differential compounds (up-regulated compound **7**), with 8 compounds common to all three groups (Figures S2a and S3a–c). Using the BCK treatment, which had a higher content of volatile compounds in the tomato leaves, as a control, enabled the analysis of differential compounds under the BP, BT, and BX treatments. In total, 20 differential compounds were identified in BCK vs. BP (up-regulated compounds **2**, down-regulated compounds **5**) and BCK vs. BT (up-regulated compounds **1**, down-regulated compounds **2**), and a total of 18 differential compounds were found in BCK vs. BX (up-regulated compounds 1, down-regulated compounds **2**), and 7 compounds were common to all three groups (Figures S2b and S3d–f).

**Figure 4:**
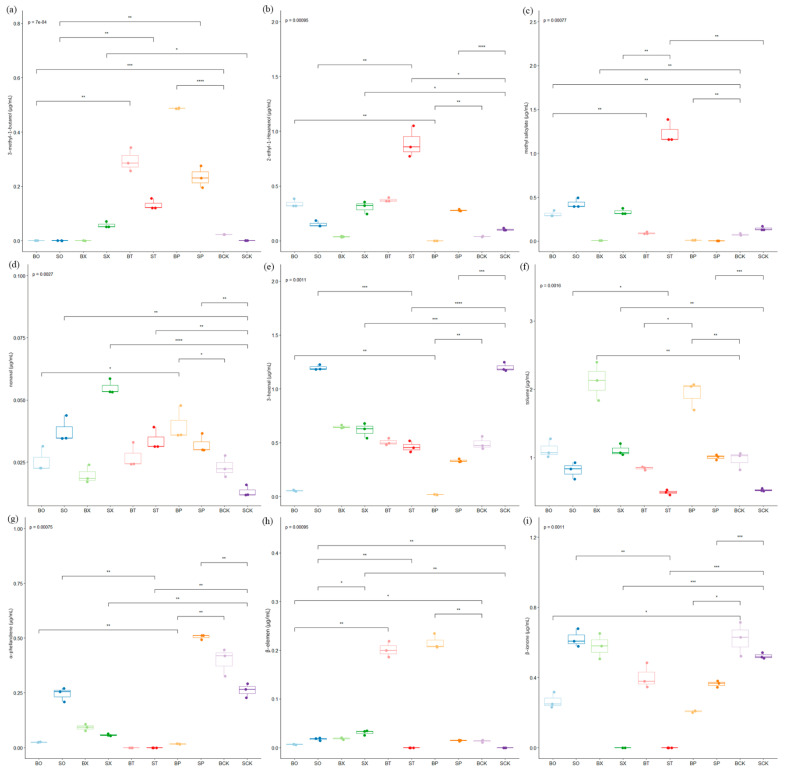
Box plots of changes in major differential volatile compounds in different treatment groups. Note: (**a**–**d**) are major differential compounds in fruits and (**e**–**i**) are major differential compounds in leaves; “*”—*p* < 0.05, “**”—*p* < 0.01, “***”—*p* < 0.001, “****”—*p* < 0.0001.

The differential compounds observed in the tomato fruits in the three comparisons included 3-methyl-1-butanol, 2-ethyl-1-hexanol, nonanal, and methyl salicylate, with significant differences noted between the treatment groups (Figure 4a–d). Notably, styrene, 3-methyl-1-butanol, and (E, E)-2,4-hexadienal were among the main up-regulated compounds, while methyl salicylate emerged as the main down-regulated compound in BCK vs. BP (Figure S3a). Among them, the 3-methyl-1-butanol content showed a significant positive correlation with (E)-2-hexen-1-ol, 2-hydroxybenzoic acidethyl ester, 3-octanone, methyl hexanoate, and 3-hexenal. Similarly, the methyl salicylate content showed a significant positive correlation with 2-ethyl-1-hexanol (Figure S4a). In addition, methyl salicylate and nonanal were significantly up-regulated compounds in both SCK vs. SO and SCK vs. SX (Figure S3b,c). The differential compounds identified in the tomato leaves comprised 3-hexenal, toluene, α-phellandrene, β-elemen, and β-ionone, with most differences being significant between treatment groups (Figure 4e–i). Among these compounds, α-phellandrene exhibited significant down-regulation in both BCK vs. BP and BCK vs. BX, and β-elemen was prominently up-regulated in BCK vs. BP and BCK vs. BT (Figure S3). β-elemen content showed a significant negative correlation with hexanal and (Z)-2-penten-1-ol (Figure S4b).

### 3.3. OAV Analysis of Different Volatile Organic Compounds

**Figure 5:**
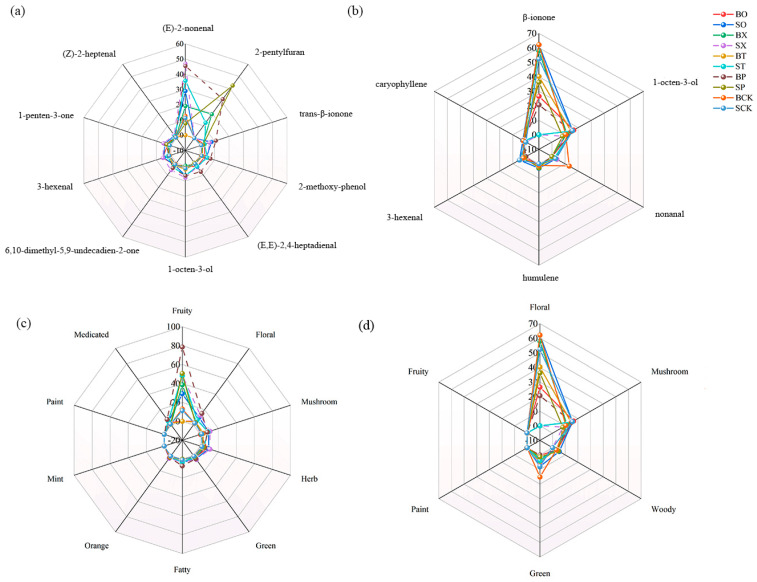
Radar fingerprints of volatiles from tomato fruits and leaves under different treatments. Note: (**a**,**c**)—fruits and (**b**,**d**)—leaves.

The top ten compounds with OAV values of ≥1 in the volatile compounds of the tomato fruits and leaves under different treatments are shown in Figure 5a,b. Under the SX treatment, the fruits’ (E)-2-nonenal, 1-octen-3-ol, 6,10-dimethyl-5,9-undecadien-2-one, 3-hexenal, and 1- penten-3-one had the highest OAV values among all treatments. The OAV values of trans-β-ionone, 2-methoxyphenol, and (E, E)-2,4-heptadienal were the highest in the fruits under the BP treatment; the OAV values of 2-pentylfuran were highest under the SP treatment. In addition, the OAV of β-ionone in the tomato leaves was greatest under all treatments, with the greatest OAV of β-ionone and nonanal in the tomato leaves under the BCK treatment and the lowest OAV of β-ionone under the BP treatment.

The compounds that contributed significantly to the aroma of the main differential substance profile of the tomato fruits included (E)-2-nonenal, trans-β-ionone, and 2-pentyl furan (Table 1). The tomato fruit and leaf odors were characterized based on OAV values of ≥1 and major differential compounds (Figure 5c,d). Floral, fruity, and fatty odor contributions were higher under the BP treatment compared to other treatments, accounting for 24.21%, 19.72%, and 11.52%, respectively, while the herb odor of the tomato fruits was more pronounced under SX than the other treatment groups; green odor percentage was higher in the BP and SX treatments. However, floral odor was most prominent in the tomato leaves, except for mushroom odor contribution, which was higher under the ST and SX treatments compared to other odors. The highest contribution of floral and green odor was observed in the BCK treatment, while the lowest contribution of floral odor was in the BP treatment group.

### 3.4. KEGG Enrichment Analysis of Volatile Compounds in Tomato Fruits and Leaves under Different Treatments

**Figure 6:**
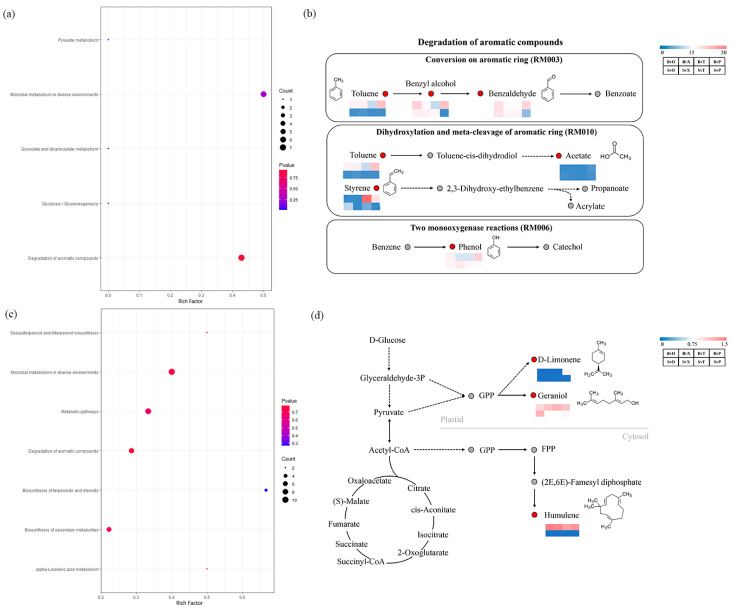
KEGG enrichment plots of volatile compounds in tomato fruits and leaves. (**a**,**c**)—KEGG bubble plots of volatile compounds in tomato fruits and leaves; (**b**)—degradation metabolic pathway plots of aromatic compounds in tomato fruits, red circles represent enriched compounds and grey circles represent non-enriched compounds; color blocks represent FC values between different groups. (**d**)—Biosynthetic pathway map of terpenoids and steroids in tomato leaves, red circles represent compounds enriched for and grey circles represent compounds not enriched for; color blocks represent FC values between different groups. Note: (**a**,**b**)—fruit and (**c**,**d**)—leaf.

KEGG enrichment analyses were conducted on the metabolic pathways of the volatile compounds in the tomato fruits and leaves. The findings revealed that the ratio of the pyruvate metabolism, glycolysis/gluconeogenesis, and glyoxylate and dicarboxylate metabolism pathways in the tomato fruits in the KEGG enrichment analyses was 0, indicating no enrichment of differential substances in these pathways (Figure 6a). Among the fruit differential volatile compounds, the degradative metabolic pathway of aromatic compounds was enriched with more volatile compounds, primarily concentrated in the three modules of aromatic ring conversion, dihydroxylation, and the intercalation of aromatic rings and two monooxygenase reactions, with a *p* value of 0.924 (Figure 6b). The content of substances in the degradation metabolic pathways of the aromatic compounds annotated under the BP treatment was higher than that of the control, suggesting that the degradation of aromatic compounds in the tomato fruits was enhanced under the BP treatment. The tomato leaves exhibited the most significant enrichment in the biosynthetic pathways of terpenoids and steroids, with the highest number of differential metabolites enriched in the microbial metabolic pathways in different environments (Figure 6c). Specifically, the metabolic pathway of terpenoids and steroids biosynthesis in the tomato leaves under the BP treatment appeared more active compared to the other treatment groups. This pathway, based on tricarboxylic acid cycle substances, synthesized humulene, d-limonene, and geraniol through the MEP (mevalonate pathway) (Figure 6d).

## 4. Discussion

### 4.1. Identification of Volatile Components in Tomato Fruits and Leaves under Different Fertilization Conditions

To address consumer demand for both the sensory and nutritional quality of tomato fruits and the efficient and rational application of organic fertilizers in orchards, we examined the impact of two organic fertilizers pelletized with different material-coated groups on the volatile compounds in tomato fruits and leaves. In this study, a total of 67 volatile compounds were identified in the tomato fruits using HS-SPME-GC-MS, with 23 volatile compounds being common across different treatments. In total, 62 volatile compounds were detected in the tomato leaves, of which 26 volatile compounds were common across the different treatments. These results indicate that the application of different organic fertilizers had a greater effect on the volatile compounds in the tomato fruits compared to the leaves. In a previous study, Zeng et al. investigated the effect of methyl salicylate treatment on the volatiles of tomato fruits stored at low temperatures, identifying a total of 37 volatile compounds through GC-O-MS analysis [20]. Similarly, Wang et al. identified 42 volatile compounds from ripe “FL 47” tomatoes using HS-SPME-GC-MS analysis. The number of compounds identified in these two studies closely resembled the findings in our investigation of tomato fruits [21]. Aldehydes were the dominant volatile compounds in the tomato fruits, whereas alcohols were prevalent in the leaves under varying fertilization conditions. The volatile compound content of the tomato fruits under the BP treatment was the highest at 35.38 μg/g, significantly higher than that of BCK. Compared with the volatile compound content of the 15 tomato varieties studied by Zhang et al. [22], Yuanwei No. 1 exhibited the highest content among the 15 varieties, resembling that of the BP-treated tomato. This suggests that the BP treatment increased the volatile compound content in the tomatoes. Fatty acids and carotenoid volatiles are key compounds affecting the flavor of tomato fruits, and C18 linoleic (18:2) and linolenic (18:3) acids serving as precursors, undergoing degradation via lipid degradation enzymes such as lipoxygenase (TomLoxC) and 13-hydroperoxide lyase (13-HPL). This process leads to the formation of corresponding short-chain C6 aldehydes, including hexanal, cis-3-hexenal, and 1-hexanol. Additionally, pentanal and trans-2-pentenal can be produced using fatty acids as precursors [23,24]. The degradation of carotenoids also produces certain aldehydes, suggesting that the substantial presence of volatile aldehydes in tomato fruits likely originates from both fatty acids and carotenoids [25]. Combined with the correlation analysis of different treatment groups with the volatile substances in tomato fruits, the content of methyl salicylate in the fruits exhibited no significant correlation with the BP and BX treatments, but showed a significant correlation with all other treatment groups, indicating that the content of methyl salicylate did not significantly increase under the BP and BX treatments.

In addition, the content of volatile compounds in the tomato fruits exhibited a significant increase in the BP and SX treatment groups compared to the two control groups, with a higher rise in alcohol content observed in the BP treatment group relative to SX. Russo et al. demonstrated that waste source fertilizers can improve tomato quality and aroma, findings that align with those of our study [26]. However, the content of volatile compounds in the tomato leaves was lower in the BP and SX treatment groups than in their respective control groups, with a more pronounced reduction observed in the BP treatment group than in the SX treatment group. Some studies have indicated that the use of organic fertilizers can also increase the chlorophyll content in leaves. Specifically, an increased magnesium (Mg) content through fertilizer application has been shown to significantly elevate the chlorophyll levels in cigar tobacco leaves, consequently enhancing plant photosynthesis and impacting the flavor components in the fruit [27]. It is inferred that the increased chlorophyll content in tomato leaves resulting from fertilizer application boosts photosynthesis in tomato plants. Consequently, the abundant amount of photosynthesis products facilitates an increase in the volatile compound content in the fruits, leading to a decrease in the volatile compound content in the leaves. Although the differences in volatile compounds between the two control groups of tomato fruits in BCK and SCK were minimal, there were some distinctions in the content of volatile compounds in the leaves. This divergence is speculated to be potentially linked to the differing environments of the two areas where the tomatoes were cultivated on a small scale. Hence, different control groups were selected for the tomatoes grown in these two areas. In terms of the volatile compound content, the BP treatment group exhibited higher levels of volatile compounds in the fruits and lower levels in the leaves compared to the control, indicating its greater efficacy when applied to tomato plants.

### 4.2. Identification of Differential Volatile Organic Compounds in Tomato Fruits and Leaves under Different Treatments

The validity of the model was confirmed by OPLS-DA and cross-validation for the analysis of the differential volatile compounds in the tomato fruits and leaves. Ten treatment groups of tomato fruit and leaf differential compounds were screened for log_2_|FC| ≥ 1 and VIP > 1. Among the top 20 volatile compounds in the tomato fruit differentials, styrene and 3-methyl-1-butanol were the up-regulated compounds with significant differences. While styrene has not been previously reported in tomato fruit volatile compounds, it is a common volatile compound known for its presence in food products, posing an aroma defect (celluloid odor). Even low concentrations can deter consumers, presenting a challenge for the food industry [28,29]. In a study by Zahra et al. [30], it was observed that styrene migrated from polystyrene packages to the foodstuffs they came into contact with, showing correlations with food characteristics such as fat content and pH levels. Therefore, it is considered that the styrene present in the tomato fruits and leaves originated from the material of the packaged organic, resulting in differences in the volatile compounds. Combined with the KEGG enrichment analysis of the different compounds, it is hypothesized that styrene is primarily involved in the metabolic pathway responsible for the degradation of aromatic compounds by microorganisms in tomato fruits, while 3-methyl-1-butanol is synthesized in tomato fruits through a complex pathway using mainly amino acids (alanine, valine, and leucine, etc.) as precursors and imparts a grassy flavor to the fruit [31,32]. Methyl salicylate, the differential compound with the highest multiplicity of differences in the tomato fruit, is produced by the methylation of salicylic acid. It is more commonly found in unripe tomato fruit, lending a minty flavor that tends to be disliked by consumers [33]. Thus, it was concluded that the different fertilization treatments mainly led to increases in the contents of styrene and 3-methyl-1-butanol compounds, while decreasing the content of methyl salicylate in the tomato fruits. Additionally, increases in the contents of β-elemen, α-phellandrene, and 1-butanol, along with a decrease in the content of 3-hexenal, were noted in the leaves.

In combination with the tomato fruit and leaf volatile compound contents, the BCK vs. BP, SCK vs. SO, and SCK vs. SX treatment groups were selected for fruit analysis, while BCK vs. BP, BCK vs. BT, and BCK vs. BX were selected for leaf analysis. Further differential compound analyses were carried out for the six treatment groups. Further differential compound analyses were conducted for these six treatment groups. In the tomato fruit BCK vs. BP, the major up-regulated compounds included styrene, 3-methyl-1-butanol, and (E, E)-2,4-hexadienal, while methyl salicylate emerged as the primary down-regulated compound. Presently, (E, E)-2,4-hexadienal is rarely documented in tomato fruit. However, volatile compounds have been identified in highbush blueberry fruit varieties (‘Primadonna’, ‘Jewel’, ‘Snowchaser’, and ‘FL02-40’) and Gabiroba (*Campomanesia xanthocarpa* O. Berg), imparting fruity flavors to the fruit [34,35]. These findings suggest that BP treatment enhances the fruity and grassy flavors in tomato fruit, facilitates the degradation of aromatic compounds, and reduces the minty flavor associated with methyl salicylate. In the comparisons of SCK vs. SO and SCK vs. SX, only methyl salicylate and nonanal exhibited significant up-regulation. Wang et al. previously identified higher levels of nonanal in tomato pericarp tissues compared to the interior of tomato fruits, aligning with our findings of nonanal in the tomato fruits, imparting an orange flavor [36]. This suggests that the SO and SX fertilizer treatments increased the orange flavor of the tomato fruits and the pungent mint flavor of the tomato fruits. Furthermore, in the tomato leaves, α-phellandrene was a significantly down-regulated compound under the BCK vs. BP and BCK vs. BX treatments, while β-elemen was a significantly up-regulated compound under the BCK vs. BP and BCK vs. BT treatments. Wei et al. analyzed the volatile aroma constituents of mango leaves with a β-elemen concentration of 827.5 μg/kg, and β-elemen was also identified in tomato leaves [37]. Shirokova et al. studied the accumulation of volatile compounds by 6-BAP (6-benzylaminopurine) on white chrysanthemum (*Anethum graveolens* L.), noting that treatment with 200 mg/L of 6-BAP promoted leaf growth and increased the α-phellandrene content [38]. The decrease in the α-phellandrene content of the tomato leaves under the BP and BX treatments observed in this study may be attributed to the reduction in metabolic compounds in the leaves due to the effect of chlorophyll in the plants.

Regarding the annotation of the metabolic pathways of the volatile compounds in the tomato fruits and leaves using a KEGG enrichment analysis, three metabolic pathways—pyruvate metabolism, glycolysis/gluconeogenesis, and glyoxylate and dicarboxylate metabolism—were notably enriched in the tomato fruits. However, the pathway ratios were zero, indicating no differential compounds. Of these, pyruvate metabolism and glyoxylate and dicarboxylate metabolism belong to sugar metabolism, while glycolysis/gluconeogenesis (the process by which glucose is converted into pyruvate and small amounts of ATP (energy) and NADH (reducing power) are produced) is a central pathway for the production of essential precursor metabolites in plants. The findings revealed that the three metabolic pathways were crucial for the volatile compounds in the tomato fruits and remained unaffected by fertilization. The volatile differential compounds in the tomato fruits exhibited greater enrichment in the aromatic compound degradation metabolic pathway. Moreover, the fold change values of the enriched compounds were higher in the BP treatment group compared to other groups. This pathway is mainly used for the microbial degradation of a wide range of compounds, including aromatic compounds in the environment [39]. Thus, it was hypothesized that fertilizer application affected the microbial degradation of aromatic compounds in the tomato fruits, which were also not favored by consumers, with a greater effect on the degradation of aromatic compounds under the BP treatment. In contrast, the biosynthetic pathways of terpenoids and steroids were notably enriched in the tomato leaves, with the fold change of the differential compounds being higher under the BP treatment than in other treatment groups. In their study of the mechanism of leaf color change in two purple-leaved tea tree varieties, Shen et al. discovered that saponins, alcohols, terpenoids, and steroid-related metabolites also underwent significant changes during the leaf discoloration process [40]. This suggests that the application of organic fertilizers impacts the coloration of tomato leaves, and is potentially linked to terpenoid and steroid metabolites. Although the biosynthetic pathways of terpenoids and steroids were more metabolically active in the tomato leaves under the BP treatment, the enriched compounds did not significantly differ from those in the BCK vs. BP comparison. Therefore, it is assumed that the BP treatment did not have significant effects on this pathway. Based on the analysis of volatile differential compounds and enrichment, the up- and down-regulated compounds in the BP treatment group of tomato fruits significantly influenced the fruit’s aroma profile.

### 4.3. Aroma Characteristics of Tomato Fruit and Leaves under Different Treatments

Numerous studies have identified volatile compounds in tomato fruits using HS-SPME-GC-MS. However, not all compounds, including differential compounds, contribute significantly to their flavor. Therefore, understanding the characteristic aroma compounds, their odor thresholds, and flavors remains crucial [41,42]. The enhanced synthesis and accumulation of these characteristic aroma compounds can improve the aroma and flavor of tomato fruit, thereby increasing consumer satisfaction. In our study, we highlighted the top ten compounds with OAV values of ≥1 in the volatile compounds of the tomato fruits and leaves under different treatments, Notably, (E)-2-nonenal exhibited the highest OAV value among all volatile compounds, with the SX-treated group showing the highest OAV value for (E)-2-nonenal, followed by the BP-treated group. Although relatively few studies have explored the volatile compound (E)-2-nonenal in tomato fruits, it boasts a high content and contributes to the fruity flavor in seedless watermelon [43], fresh jujube [44], and peach fruits [45]. It was evident that the aroma of the tomato fruit significantly increased under both the SX and BP treatments and was higher than the control. Following (E)-2-nonenal, 2-pentylfuran exhibited the second-highest OAV, with the highest observed under the SP treatment, followed by the BP treatment group. Buttery et al. demonstrated that 2-pentylfuran was present in trace amounts in fresh tomatoes; 2-pentylfuran also had a high content and aroma contribution in the identification of volatile compounds in seedless watermelon by Beaulieu et al. [43,46]. Moreover, the OAV value of trans-β-ionone in the tomato fruits under the BP treatment was higher than that of other treatment groups. Trans-β-ionone, a C13 volatile, imparts a floral aroma and is primarily produced through the catalytic cleavage of carotenoids by the dioxygenases LeCCD1A and LeCCD1B, thereby enhancing the flavor acceptance of tomatoes [47]. Among the remaining compounds with an OAV of ≥ 1, 1-penten-3-one and 3-hexenol originated from fatty-acid-derived volatiles, contributing to the fruity flavor of the fruit, while 6,10-dimethyl-5,9-undecadien-2-one derived from C13-norbornadiene aroma substances imparted a floral flavor to the fruit [48]. The results showed that the SX, BP, and SP treatments increased the fruity and floral flavors of the tomato fruits to varying degrees. In the leaves, the OAV value of β-ionone was maximum in the treated group and minimum in the BP-treated group, conferring fruity flavor to the fruits. It is believed that the metabolites produced by leaf photosynthesis for fruit growth and development caused an increase in the fruit flavor and a decrease in leaf β-ionone in the BP treatment group.

Combining the OAV values of different fertilizer treatment groups for various substances and volatile compounds with an OAV of ≥ 1 enables understanding the flavor of the tomato fruits and leaves in the different treatment groups. It is evident that, under the BP treatment, the tomato fruit aroma, floral aroma, green odor, and fat aroma were higher compared to other treatment groups, while the aroma in the leaves was poor. Substances influencing the flavor of the tomato fruits under this treatment primarily included (E)-2-nonenal, 2-pentylfuran, trans-β-ionone, 1-penten-3-one, (E, E)-2,4-hexadienal, and 3-hexenol (fruity, floral, and herbaceous odors), (E, E)-2,4-heptadienal (fatty odors), and hexanal (green odors) [49]. However, under the BP treatment, one medicinal odor was higher than the other treatment groups, mainly derived from 2-methoxyphenol. In the argument presented by Distefano et al., it was shown that an enigmatic biosynthetic pathway leads to the synthesis of 2-methoxyphenol, imparting a medicinal flavor [50]. However, the treatment of tomato plants with the AM fungus increased fruit 2-methoxyphenol, as shown in the study by Hart et al. [51]. This increase may be attributed to the BP treatment group affecting microorganisms, leading to an elevation in 2-methoxyphenol. However, its OAV value was low compared to that of other flavoring substances and accounted for a very small percentage of the total, thus exerting only a minor effect on the flavor of the tomato fruits. BP treatment uses polyurethane as the coating material, which is a high-strength, wear-resistant substance widely used in medicine, food, agriculture, and cosmetics. Its effects on soil are primarily reflected in enhancing soil texture, improving plant growth rate, and preventing water evaporation and environmental pollution. The vegetable oil polyurethane-coated organic fertilizer used in this experiment is biodegradable and will not cause secondary soil pollution [52].

## 5. Conclusions

In summary, in terms of the volatile compound contents of tomato fruits and leaves, the BP treatment group exhibited a higher volatile compound content in the fruits, measuring 35.38 μg/g, compared to the other treatment groups, while the volatile compound content in the leaves was lower. An analysis of the differential compounds showed that styrene, 3-methyl-1-butanol, and (E, E)-2,4-hexadienal, etc., were the major up-regulated compounds, whereas methyl salicylate was the major down-regulated compound in the tomato fruit BCK vs. BP. Additionally, there was a reduction in the content of α-phellandrene in the tomato leaves. The results of the KEGG enrichment analysis indicated that fertilizer application affected the degradation pathways of the aromatic compounds in the tomato fruits under BP treatment, while the metabolic pathways of leaf terpenoids and steroids were metabolically active. In addition, an analysis of the tomato fruit differential compounds and compounds with an OAV of ≥ 1 by the OAV values of the characteristic aroma compounds identified the key compounds affecting the flavor of the tomato fruits under the BP treatment, mainly (E)-2-nonenal, 2-pentylfuran, trans-β-ionone, 1-penten-3-one, (E, E)-2,4-hexadienal, and 3-hexenol (fruity, floral, and herbaceous odors), (E, E)-2,4-heptadienal (fatty odor), and hexanal (green odor). In conclusion, our investigation into the impacts of various granulated organic fertilizers and coating materials on tomato fruit flavor revealed that the use of granulated large-grain organic fertilizers coated with polyurethane (pozzolanic + small-grain oil-coated + 2% paraffinic + 4% polyurethane) resulted in a significant increase in the volatile compound content in tomato fruits, thereby enhancing their aroma. This formulation proved to be the most suitable for orchard application. The application of granulated and coated organic fertilizers reduces seepage losses and maintains the balance and sustainability of the soil ecosystem, laying the foundation for their use in artificial orchards. However, our study did not delve into the influence of the mineral elements in fertilizers on volatile compounds and tomato fruit and leaf yield, nor did it validate the proposed link between changes in the volatile compound contents between leaves and fruits. Future research endeavors should focus on optimizing the mineral content and composition of organic fertilizers, as well as refining granulation and coating techniques, to offer a solid scientific foundation for their commercial application.

## Figures and Tables

**Table 1:** Differential compounds and aroma contribution of tomato fruits and leaves.

CAS Number	Volatile Compounds	Odor Threshold	Odor Descriptor	OAV									
				BO	SO	BX	SX	BT	ST	BP	SP	BCK	SCK
Fruit													
Alcohols													
123-51-3	3-methyl-1-butanol	4	green	0	0	0	0.0144	0.0739	0.0332	0.1218	0.0585	0.0057	0
928-95-0	(E)-2-hexen-1-ol	2	herb, green	0	0.0190	0	0.0886	0	0	0	0	0	0.0152
104-76-7	2-ethyl-1-hexanol	-											
589-35-5	3-methyl-1-pentanol	-											
1569-60-4	6-methyl-5-hepten-2-ol	-											
Aldehyde													
66-25-1	hexanal	4.5	green	1.4994	2.3263	0.5139	2.5910	0.4701	0.8764	3.1479	1.3680	0.3621	0.4279
142-83-6	(E, E)-2,4-hexadienal	1.6	fruity	0	0.2583	0.0832	0	0.0519	0	0.0728	0	0.0360	0
124-19-6	nonanal	0.015	orange	1.7016	2.5142	1.3232	3.6720	1.8138	2.2606	2.6607	2.1430	1.5384	0.8796
18829-56-6	(E)-2-nonenal	0.00017	fruity	0	28.9365	19.1148	46.9399	0	35.6843	45.4654	7.8302	12.7064	11.3064
36431-60-4	5-ethylcyclopent-1-enecarboxaldehyde	-	-										
90-02-8	2-hydroxy- benzaldehyde	-	-										
100-52-7	benzaldehyde	350	Almond, sugar	0.0007	0.0008	0.0002	0.0004	0.0002	0.0004	0.0009	0.0001	0.0001	0.0001
79-77-6	trans-β-ionone	0.000007	Floral	3.1765	7.9833	1.8752	6.6741	1.7288	3.1551	10.9733	2.1164	1.0195	1.0627
4312-99-6	1-octen-3-one	0.03	Mushroom	0	2.9221	0.9377	2.8924	0.9861	2.1328	1.2489	0.5351	0.8621	0
Terpene													
29050-33-7	(+)-4-carene	-	-										
138-86-3	limonene	0.41	Fruity, fresh	0	0	0	0.0354	0	0.0115	0	0.1519	0.0348	0.0381
100-42-5	styrene	0.12	-	0	0.1533	0	0	0.5717	0.0623	0.1345	0	0.0208	0.1541
535-77-3	β-cymene	-	-										
555-10-2	β-phellandrene	-	Citrus										
Lipid													
123-66-0	ethyl hexanoate	0.008	Fruity	0	0	0	1.8675	0	0	1.3374	0	0.3123	0
540-18-1	pentyl butanoate	1.3	-										
119-36-8	methyl salicylate	40	Mint	0.0077	0.0107	0.0001	0.0084	0.0023	0.0309	0.0003	0.0001	0.0019	0.0036
Heterocyclic class													
3777-69-3	2-pentylfuran	0.0048	Fruity	0	0	19.4065	0	0	12.4980	31.6386	42.6739	0	0
Aromatic hydrocarbon													
108-88-3	toluene	6	Paint	0.0069	0.0079	0.0080	0.0184	0.0021	0.0038	0.0227	0.0072	0.0019	0
Leaf													
Alcohols													
71-36-3	1-butanol	450	-	0	0	0	0.0002	0	0.0002	0.0003	0.0002	0.0003	0.0004
928-97-2	(E)-3-hexen-1-ol	1	-	0.0195	0.0209	0.0160	0.0132	0.0290	0.0162	0.0717	0.0170	0.0247	0.0212
556-82-1	4-methyl-1-pentanol	820	-	0.0001	0.0001	0.0001	0.0001	0.0001	0.0001	0.0001	0.0001	0.0001	0.0001
1576-95-0	(Z)-2-penten-1-ol	40	-	0.0026	0.0019	0.0015	0.0014	0.0009	0.0018	0.0008	0.0019	0.0027	0.0027
928-95-0	(E)-2-hexen-1-ol	2	-	0.7117	0.5396	0.6279	0.4783	0	0.6579	0.2478	0.5717	1.0607	0
Aldehyde													
66-25-1	hexanal	4.5	Green	0.0223	0.0165	0.0112	0.0170	0.0098	0.0146	0	0.0067	0.0152	0.0168
6728-26-3	3-hexenal	0.25	Green	0.2197	4.7897	2.5946	2.4679	2.0264	1.8478	0.0711	1.3323	1.9721	4.8035
142-83-6	(E, E)-2,4-hexadienal	1.6	Fruity	0.2404	0.2415	0.1512	0.2607	0.1257	0.1309	0.0475	0.0559	0.1293	0.2114
124-19-6	nonanal	0.015	Green	0	3.0767	0	3.7299	2.5317	1.9216	0	0	13.1816	2.8124
67-47-0	5-hydroxymethylfurfural	-	-										
Ketone													
30086-02-3	(E, E)-3,5-octadien-2-one	0.3	-	0.0591	0	0.1033	0	0	0	0.0774	0	0.1240	0
14901-07-6	β-ionone	0.01	Floral	26.5547	62.0895	57.9328	0	40.2516	0	20.6896	36.3033	62.1466	52.3072
Terpene													
515-13-9	β-elemen	-	-										
99-83-2	α-phellandrene	-	-										
138-86-3	d-limonene	0.41	-	0	0.5260	0	0.9314	0	0.5925	0.4064	0.9831	0.7827	0
100-42-5	styrene	0.12	-	0.8402	0.9897	0.2371	0.5613	0.2261	0.6125	0.6530	0.6482	0.7377	0.9803
1139-30-6	caryophyllene oxide	-	-										
6753-98-6	humulene	0.16	-	1.5944	3.0284	1.5389	0	1.2869	1.1705	1.4024	2.8001	1.0571	0
29050-33-7	(+)-4-carene	-	-										
Lipid													
84-74-2	dibutyl phthalate	-	-										
Aromatic hydrocarbon													
535-77-3	1-methyl-3-(1-methylethyl)- benzene	-	-										
108-88-3	toluene	6	Paint	0.1865	0.1358	0.3539	0.1845	0.1405	0.0817	0.3225	0.1676	0.1617	0.0872
Phenols													
96-76-4	2,4-bis(1,1-dimethylethyl)- phenol	-	-										
97-53-0	eugenol	0.5	Sweet	0.0622	0.0545	0.0336	0	0.0208	0.0542	0.0403	0.0224	0.02688	0.0272

## Data Availability

Data of this project have been deposited in Mendeley Data. Repository name: Effects of Pelletized and Coated Organic Fertilizers on Flavor Compounds of Tomato Fruits and Leaves. https://data.mendeley.com/datasets/w9dp7dn37g/1. Further inquiries can be directed to the corresponding authors.

## Supplementary Materials

The following supporting information can be downloaded at: https://www.mdpi.com/article/10.3390/foods13111653/s1, Figure S1: Thermogram of correlation between different treatment groups and volatile substances in tomato fruits and leaves; Figure S2: Wayne plots of compounds differing between treatment groups; Figure S3: Volcano plots of different volatile compounds among different treatment groups. Tomato fruits: (**a**) BCK vs. BP; (**b**) SCK vs. SO; (**c**) SCK vs. SX; tomato leaves: (**d**) BCK vs. BP; (**e**) BCK vs. BT; (**f**) BCK vs. BX; Figure S4: Heatmap of volatile compounds in tomato fruit and leaf.; Table S1: Results of organic fertilizer sampling and testing.
