# Supplementary material for: Effects of Pelletized and Coated Organic Fertilizers on Flavor Compounds of Tomato Fruits and Leaves
Source: Foods. 2024 May 25;13(11):1653. doi: 10.3390/foods13111653 (PMC11171810; doi:10.3390/foods13111653)
Supplement: Supplementary file 1 [file foods-13-01653-s001.zip › foods-2996713-supplementary.pdf]

Table S1. Results of organic fertilizer sampling and testing

Sample No.	Sample Status	Total nitrogen (%)	K_2O (%)	P_2O_5(%)	pH	organic matter (%)	Cd(mg/kg)	Cr(mg/kg)	Pb(mg/kg)	As(mg/kg)	Hg(mg/kg)
B	granule	2.74	3.50	3.73	7.0	55.8	0.52	17.9	8.53	6.76	0.031
S	granule	2.52	3.26	3.51	7.2	55.7	0.55	21.6	8.49	7.18	0.030

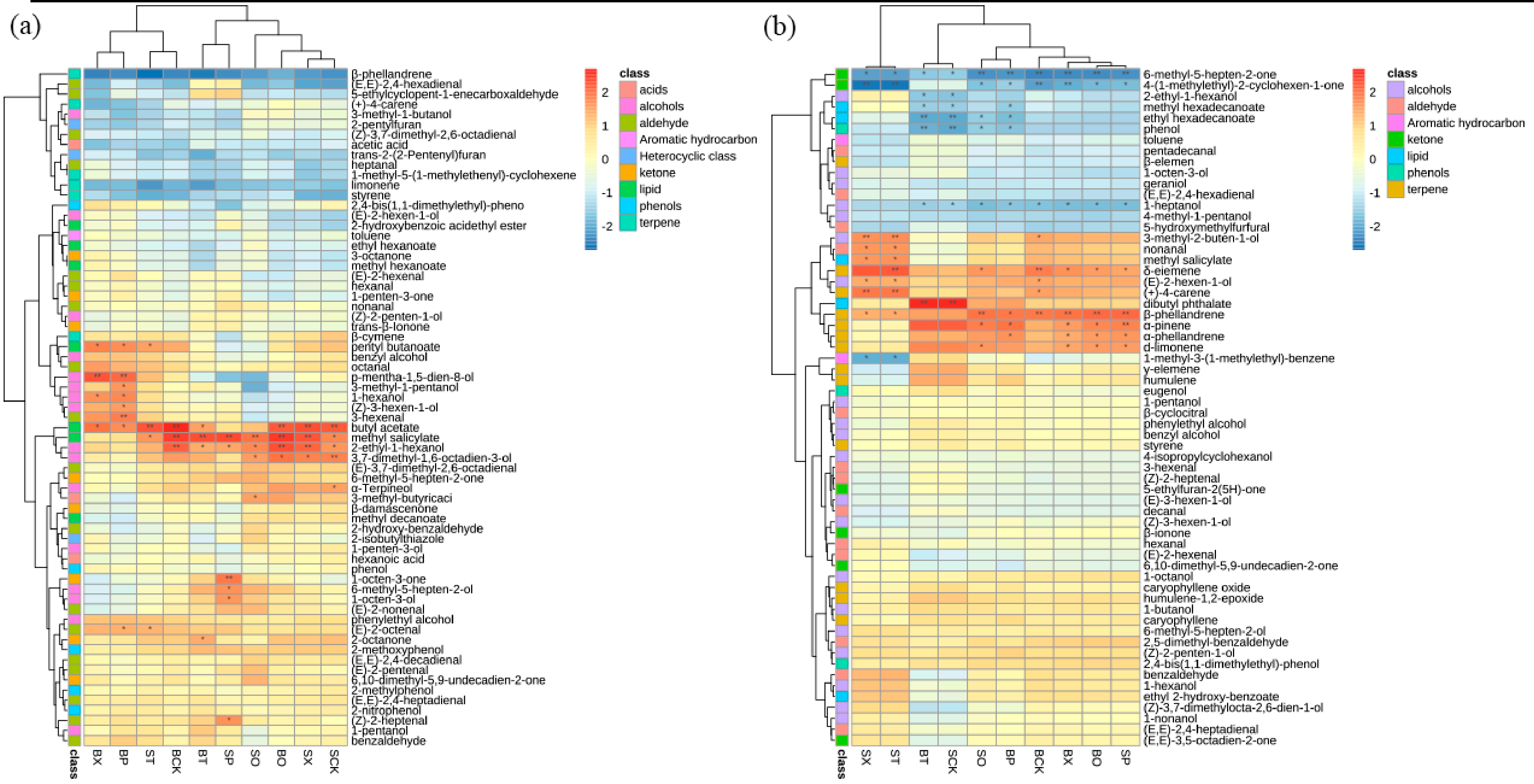

Note: a - fruit; b - leaves

Figure S1. Thermogram of correlation between different treatment groups and volatile substances in tomato fruits and leaves. * $p < 0.05$; ** $p < 0.01$.

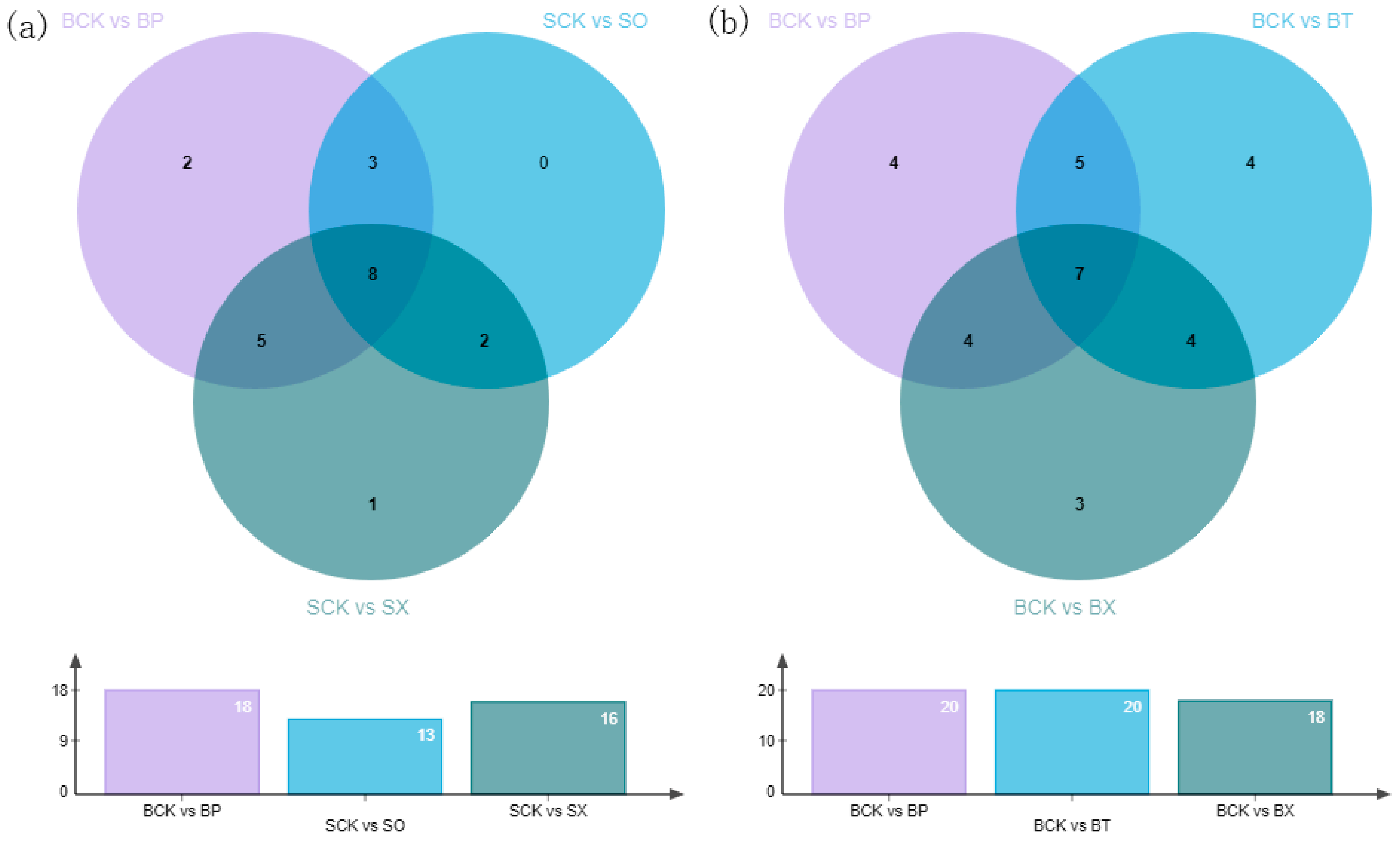

Figure S2. Wayne plots of compounds differing between treatment groups. a - fruit; b – leaf.

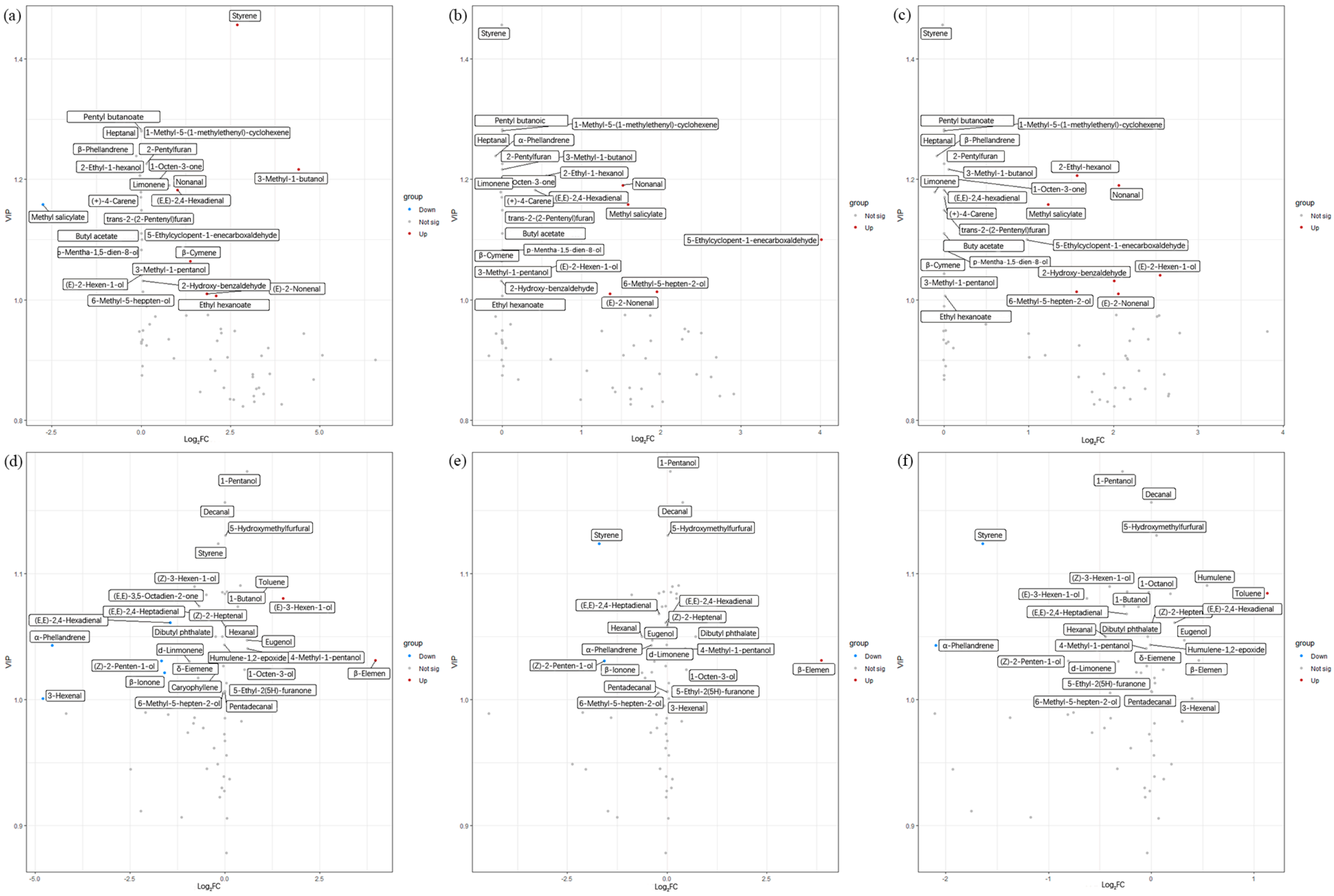

Figure S3. Volcano plots of different volatile compounds among different treatment groups. Tomato fruits: a-BCK vs BP; b-SCK vs SO; c-SCK vs SX; tomato leaves: d-BCK vs BP; e-BCK vs BT; f-BCK vs BX.

Note: a-fruit; b-leaf; arrows indicate that the order of compounds in the horizontal coordinate goes from left to right in line with the vertical coordinate from top to bottom.

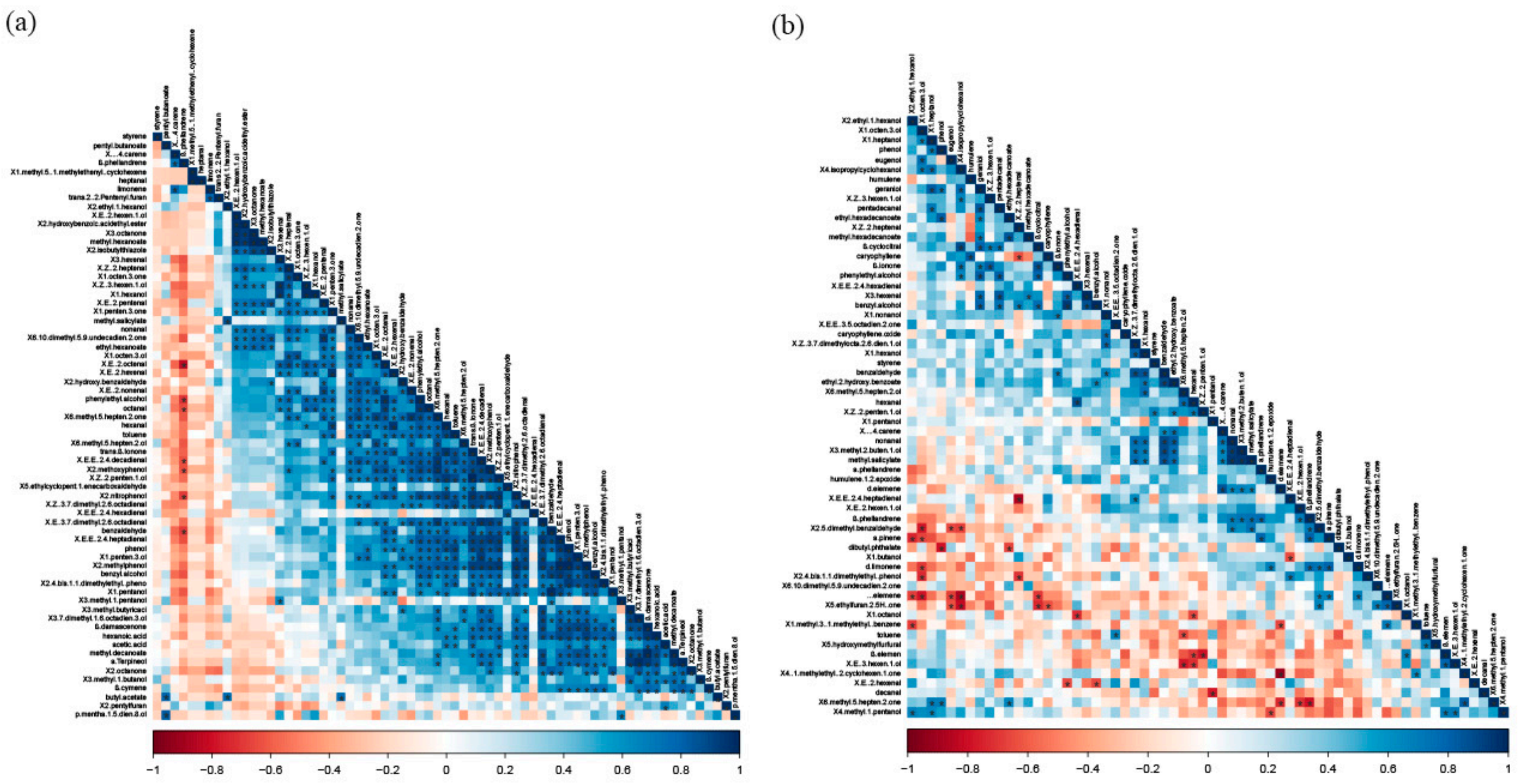

Figure S4. Heatmap of volatile compounds in tomato fruit and leaf. a - fruit; b – leaf.
